# Serum circulating cell-free messenger RNA profile response to risdiplam treatment in adult patients with late-onset spinal muscular atrophy

**DOI:** 10.1093/braincomms/fcag352

**Published:** 2026-09-25

**Authors:** Markus Leo, Linda-Isabell Schmitt, Kai Christine Liebig, Stefanie Hezel, Svenja Neuhoff, Andreas Roos, Tobias Ruck, Christoph Kleinschnitz, Tim Hagenacker

**Affiliations:** Department of Neurology, Center for Translational Neuro- and Behavioral Sciences (C-TNBS), University Medicine Essen, Essen 45147, Germany; Department of Neurology, Center for Translational Neuro- and Behavioral Sciences (C-TNBS), University Medicine Essen, Essen 45147, Germany; Department of Neurology, Center for Translational Neuro- and Behavioral Sciences (C-TNBS), University Medicine Essen, Essen 45147, Germany; Department of Neurology, Center for Translational Neuro- and Behavioral Sciences (C-TNBS), University Medicine Essen, Essen 45147, Germany; Department of Neurology, Center for Translational Neuro- and Behavioral Sciences (C-TNBS), University Medicine Essen, Essen 45147, Germany; Department of Neurology, Ruhr University Bochum, BG University Hospital Bergmannsheil, Bochum 44789, Germany; BG University Hospital Bergmannsheil, Heimer Institute for Muscle Research, Bochum 44789, Germany; Department of Neuropediatrics, Centre for Neuromuscular Disorders in Children, University Children’s Hospital Essen, University Medicine Essen, Essen 45147, Germany; Department of Neurology, Ruhr University Bochum, BG University Hospital Bergmannsheil, Bochum 44789, Germany; BG University Hospital Bergmannsheil, Heimer Institute for Muscle Research, Bochum 44789, Germany; Department of Neurology, Center for Translational Neuro- and Behavioral Sciences (C-TNBS), University Medicine Essen, Essen 45147, Germany; Department of Neurology, Center for Translational Neuro- and Behavioral Sciences (C-TNBS), University Medicine Essen, Essen 45147, Germany

**Keywords:** circulating mRNA, cfRNA, biomarker, neuromuscular disorders, liquid biopsy

## Abstract

SMN-enhancing therapies have improved outcomes in adult late-onset spinal muscular atrophy (loSMA), but minimally invasive biomarkers that reflect systemic disease and treatment effects are lacking. In this retrospective cohort study, we profiled circulating cell-free messenger RNA (ccfmRNA) in serum samples from 17 adults with loSMA type 2 (*n* = 14) and type 3 (*n* = 3) and healthy controls (*n* = 12) using the NanoString neuroimmunology panel before (T0) and after 8–10 (T1) and 12–14 (T2) months of risdiplam treatment. A separate exploratory subgroup of patients switching from nusinersen to risdiplam (*n* = 7) was analysed at comparable time points. Differential ccfmRNA expression, gene ontology and network analyses were performed. At baseline, adult loSMA showed a distinct inflammatory and stress-related ccfmRNA signature and reduced expression of selected glial- and vascular-associated transcripts compared to controls. Risdiplam partially normalized immune and proteostasis transcripts, whereas glial and vascular modules remained persistently dysregulated. Exploratory subgroup analyses suggested larger transcriptional deviations in type 2 than in type 3, although these findings are limited by small sample size. In switcher patients, additional transcriptional changes involving epigenetic- and lipid-associated pathways were observed and should be regarded as hypothesis-generating. Serum ccfmRNA profiles distinguish adult loSMA from controls and capture risdiplam-associated molecular responses. These findings support the concept that serum ccfmRNA profiling may provide a useful exploratory framework for studying disease-associated and treatment-associated molecular changes in adult loSMA.

## Teaser

Leo *et al*. report that serum cell-free messenger ribonucleic acid distinguishes adult spinal muscular atrophy from controls and shifts with risdiplam. Immune and stress-related signals fell towards control levels, whereas transcripts linked to glial and vascular support stayed abnormal. This supports a biomarker to monitor treatment and reveal residual pathology.

## Graphical Abstract

**Graphical Abstract:**
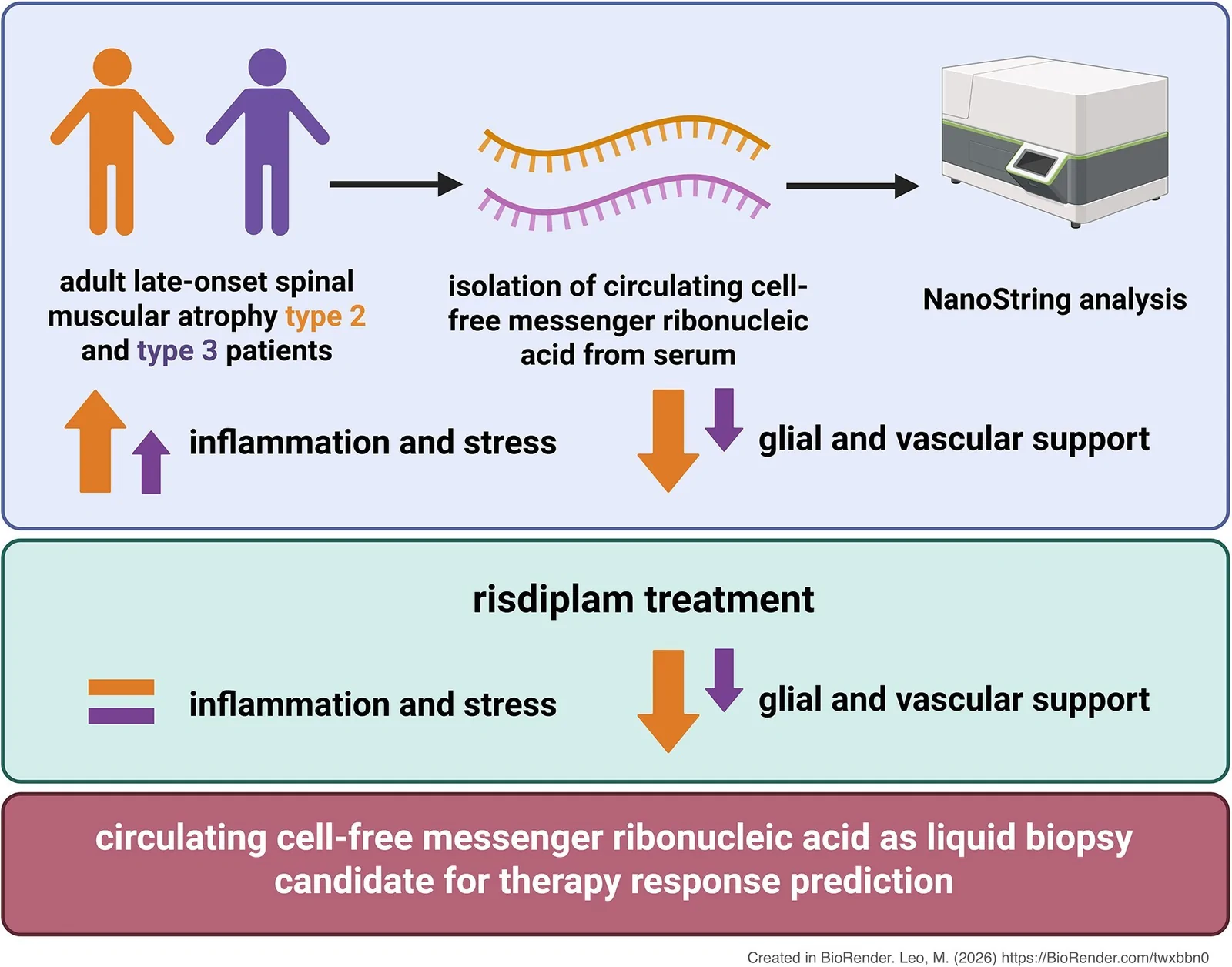

## Introduction

Spinal muscular atrophy (SMA) is a rare, genetic neurodegenerative disorder characterized by progressive muscle weakness and atrophy, resulting from the loss of spinal motor neurons. It is caused by deletions or mutations in the *survival of motoneuron 1* (*SMN1*) gene, leading to a deficiency of functional SMN protein.^1,2^ The paralog gene *SMN2* produces limited amounts of functional SMN protein due to alternative splicing and cannot fully compensate for the loss of *SMN1*. However, *SMN2* acts as a disease-modifying gene. The clinical severity of SMA phenotypes varies widely and is negatively correlated with the number of *SMN2* copies.^2-4^

The therapeutic landscape of SMA has undergone significant changes in recent years with the approval of three SMN-enhancing treatments: the antisense oligonucleotide nusinersen, the oral splicing modulator risdiplam and the gene replacement therapy onasemnogene abeparvovec.^5-10^ These interventions have improved outcomes, particularly when administered early in the disease course. However, therapeutic responses in adult patients with late-onset spinal muscular atrophy (loSMA), who often begin treatment after substantial, irreversible MN loss, remain variable and frequently limited.^10-13^ As such, there is a growing need for strategies that complement SMN-targeting therapies and for reliable molecular biomarkers to monitor disease progression and treatment response.

Circulating cell-free ribonucleic acids (ccfRNAs), including messenger RNA (mRNA) and microRNA (miRNA), are detectable in various body fluids, such as blood (serum) and cerebrospinal fluid, and have emerged as promising non-invasive biomarkers for a range of neurological and systemic disorders.^14-18^ These molecules may originate from cellular stress, degeneration or active secretion, and can reflect ongoing pathophysiological processes. In neurodegenerative diseases such as amyotrophic lateral sclerosis and Alzheimer’s disease, alterations in ccfRNA profiles have been associated with disease severity or therapy response. Their utility as diagnostic or prognostic indicators in adult loSMA remains underexplored.

In a recent study, we applied high-throughput transcriptomic analysis to serum samples from adult loSMA patients undergoing treatment with nusinersen using a NanoString Technology approach. We identified distinct gene expression changes that correlated with clinical phenotypes and treatment responsiveness, highlighting the potential of ccfmRNA as a source of SMN-independent biomarkers and therapeutic targets.^19^

In the present study, we extend this approach to adult loSMA patients treated with risdiplam, to explore whether serum ccfmRNA profiles capture treatment-associated molecular changes under this alternative SMN-enhancing therapy. By analysing ccfmRNA profiles before and under risdiplam treatment, we aimed to identify candidate transcriptional patterns associated with adult loSMA and their longitudinal modulation under therapy. Ultimately, these data may contribute, although representing a more exploratory and hypothesis-generating approach, to the development of more individualized treatment strategies and expand the set of biomarkers available for clinical monitoring and therapeutic outcome prediction in adult loSMA.

## Materials and methods

### Serum samples of loSMA patients and healthy control individuals

Based on retrospective sample availability, serum samples of 17 adult loSMA patients (male and female, age 20–58 years) with 5q-SMA (type 2 or 3) treated with risdiplam and 12 age-matched healthy control individuals (male or female) were selected. Additionally, seven nusinersen-to-risdiplam switchers (male and female, age 25–41 years; one was excluded due to isolation problems), treated with nusinersen for 12–51 months (mean: 34 months) before switching to risdiplam, were analysed (Table 1).

**Table 1:** Demographic data of included SMA patients

Characteristic	Category/Statistic	Value	*N* available	Missing/Note
**Sex**	Male	14 (60.9%)	23	
	Female	9 (39.1%)	23	
**SMA type**	Type 2	15 (65.2%)	23	
	Type 3	8 (34.8%)	23	
**SMN2 copy number**	Two copies	1 (4.3%)	23	
	Three copies	19 (82.6%)	23	
	Four copies	3 (13.0%)	23	
**Motor status**	Non-sitter	14 (60.9%)	23	
	Sitter	8 (34.8%)	23	
	Walker	1 (4.3%)	23	
**Spondylodesis**	Yes	12 (52.2%)	23	
	No	11 (47.8%)	23	
**Non-invasive ventilation**	Yes	7 (30.4%)	23	
	No	16 (69.6%)	23	
**Prior treatment with nusinersen**	Yes	7 (30.4%)	23	
	No	16 (69.6%)	23	
**Duration of prior nusinersen treatment (years)**	Mean ± SD	2.51 ± 1.63	23	
	Median (IQR)	3 (3.3)	23	
	Range	0.5–4.2	23	
**Age (years)**	Mean ± SD	36.00 ± 9.77	23	
	Median (IQR)	36.00 ± 9.77	23	
	Range	20–58	23	
**Disease duration (years)**	Mean ± SD	34.22 ± 9.20	23	
	Median (IQR)	34.1 (13.75)	23	
	Range	19–57	23	
**HFSME score at baseline**	Mean ± SD	3.77 ± 8.75	22	one baseline value not available
	Median (IQR)	1 (4.0)	22	
	Range	0–42	22	
**RULM score at baseline**	Mean ± SD	10.65 ± 8.03	22	one baseline value not available
	Median (IQR)	10.65 ± 8.03	22	
	Range	0–36	22	

Serum samples were obtained from adult loSMA patients at three different times: prior to treatment (T0), 8–10 months afterward (T1) and 12–14 months following the initial risdiplam dose (T2). Late-onset SMA patients were categorized based on their therapeutic response using the Hammersmith Functional Motor Scale Expanded. They were classified as responders (R) if they showed a score increase of at least two points, or non-responders.^20,21^

### Ethics approval and consent to participate

Study approval was obtained from the University Duisburg-Essen ethics committee (approval number 18-8285-BO). Informed consent was obtained from all subjects involved in the study.

### RNA extraction

RNA was extracted from 500 µl of patients’ serum using the Maxwell RSC miRNA from Plasma or Serum kit, which efficiently isolates high-quality, amplifiable total RNA—including small fragments—from mammalian serum or plasma samples (Promega, WI, USA). The purified RNA was eluted in 50 µl of RNase-free water and stored at −80°C. Prior to analysis, RNA concentration was measured with the Qubit Fluorometric Quantification (Thermo Fisher Scientific, MA, USA), following the manufacturer’s guidelines for the RNA broad-range assay kit. Due to the limited free RNA quantity in human serum, the maximum available volume was utilized for subsequent analysis.

### nCounter CodeSet design and expression analysis

The ‘neuroimmunology’ nCounter panel incorporates a cell-typing function that enables the quantification of neurons, astrocytes, microglia, oligodendrocytes and endothelial cells. It comprises 770 genes, offering a thorough evaluation of 23 related pathways and 30 reference genes. Hybridizations were conducted using the high-sensitivity protocol on the nCounter Prep-Station. Subsequently, post-hybridization processing was executed with the nCounter MAX/FLEX System (NanoString), and the cartridge was scanned utilizing the Digital Analyser (NanoString). The cartridge was ultimately read at maximum sensitivity (555 FOV).

### NanoString data processing

Initially, the counts obtained for the positive control probe sets were analysed. The raw NanoString counts for each gene underwent technical factorial normalization by subtracting the mean counts plus twice the standard deviation derived from the CodeSet’s inherent negative controls. Subsequently, a biological normalization was implemented utilizing the included RNA reference genes. The raw counts, normalized values and *z*-scores are available in Supplementary Table 1.

### *In silico* analysis

*In silico* analysis tools were employed to detect individual transcription differences in patients. A hierarchically clustered heatmap was generated using SRplot. To analyse the differentially expressed genes, a volcano plot was created, with discrimination based on a significance threshold of *P* < 0.05 and a minimum log2 fold change of 0.5.

Gene ontology (GO) terms were identified using Cytoscape and the BinGO app (different GO terms can be found in Supplementary Table 2). A network with interaction partners was created using Cytoscape and NedREX Diamond.

Targets for each network cluster were identified using NeDREX and the drug analysis tool as described in Sadegh *et al*.^22^ In short, genes from each different cluster were seeded within the NeDREX app and the DIseAse MOdule Detection (DIAMOnD) algorithm was used. First, a new network from the NeDRex Database was imported into Cytoscape (File → Import → Network from public Database → Data Source: NeDREX: network query from NedRexDB). Afterwards, the significantly changed genes were selected from the database and run with the DIAMOnD tool. No neighbour proteins were added. Further, the drug prioritization tool with the trust rank tool was added. The nodes were weighted with a damping factor of 0.85 and only approved and direct drugs were listed.

Lastly, targets for each network were identified using NedREX and the drug analysis tool.

### Statistical analysis

Experimental results were analysed statistically using GraphPad Prism 9.4.1 software. Initially, all data were tested for normal distribution with the Shapiro–Wilk test. Depending on the data and experiment, analysis was performed using either one-way ANOVA with Holm–Sidak’s multiple comparison test (for three groups with normal distribution) or Kruskal–Wallis test with Dunn’s multiple comparison test (for three groups without normal distribution). When samples from the same patients were taken at least three time points, a within-subjects (repeated measures) analysis was applied. The specific test and the corresponding *P*-, *F*-, or *H*-values are detailed in Supplementary Table 3. All figures display *P*-values. Significance levels were set as follows: *P < 0.05, **P < 0.01, ***P < 0.001. Data are presented as mean ± standard deviation (SD).

## Results

### Serum ccfmRNA profile alterations distinguish adult loSMA from healthy individuals

**Figure 1:**
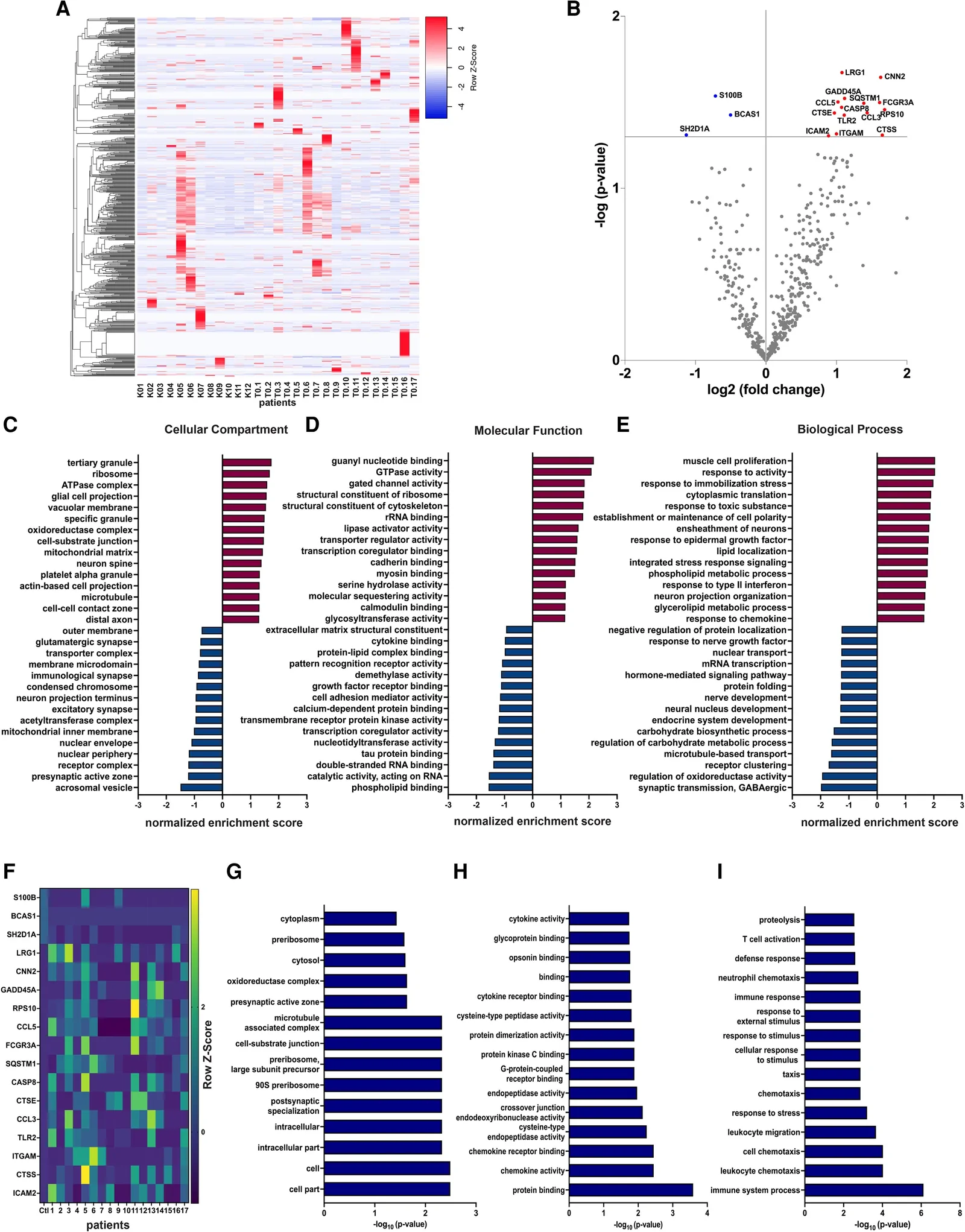
**Comparison of untreated adult loSMA patient samples to healthy individuals.** (**A)** Heat map of all measured transcripts of the serum of untreated adult loSMA patients (17) and controls (12) using directional McQuitty clustering. *Z*-scores are plotted for each patient. (**B)** Volcano plot illustrating the distribution of all transcripts (770). For each gene, the fold change was calculated by dividing the mean transcript abundance in untreated patients with loSMA by the mean transcript abundance in controls. The corresponding *P*-value was calculated from the individual patient values using a two-sided, unpaired Student’s *t*-test. Statistical significance is displayed as −log(*P*) and the horizontal line at −log(*P*) = 1.301 represents the nominal unadjusted significance threshold of *P* = 0.05. Significantly altered transcripts (above the horizontal line) are categorized as upregulated or downregulated. Top ten GO terms cellular components (**C**), molecular functions (**D**) and biological processes (**E**) identified using GSEA. (**F)** Heat map (*z*-scores) of all significantly regulated transcripts of the serum of untreated adult loSMA patients and pooled controls. Top ten GO terms of cellular components (**G**), molecular functions (**H**) and biological processes (**I**) identified using ORA. *n* = 12 controls, *n* = 17 SMA patients.

Nanostring analysis was used to measure ccfmRNA in serum samples of adult loSMA and healthy control individuals. Hierarchical clustering of all measured transcripts visualized as a heat map, revealed significant alterations in ccfmRNA expression between adult loSMA and healthy individuals (Fig. 1A). A volcano plot was constructed to visualize the distribution of all quantified transcripts, using log2 FC plotted against −log10 adjusted *P*-values (|log_2_FC| ≳ 0.5; −log_10_*P* > 1) (Fig. 1B). Overall, 17 transcripts were significantly changed, with 14 being upregulated including *C-C Motif Chemokine Ligand 3* (*CCL3*) *CCL5, Toll Like Receptor 2* (*TLR2), Intercellular Adhesion Molecule 2* (*CAM2*)*, Integrin Alpha M* (*ITGAM*)*, Cathepsin S* (*CTSS*)*, Ribosomal Protein S10* (*RPS10*)*, Cathepsin E* (*CTSE*)*, Caspase 8* (*CASP8*)*, Fc Gamma Receptor IIIa* (*FCGR3A), Sequestosome 1* (*SQSTM1*)*, Growth Arrest and DNA Damage Inducible Alpha* (*GADD45A*)*, Calponin 2* (*CNN2*)*, Leucine Rich Alpha-2-Glycoprotein 1* (*LRG1*) and three downregulated including *S100 Calcium Binding Protein B* (*S100B*), *Brain Enriched Myelin Associated Protein 1* (*BCAS1*) and *SH2 Domain Containing* 1A (*SH2D1A*).

A gene set enrichment analysis (GSEA) analysis was performed to identify the top ten up- and downregulated GO terms in three categories: cellular components (CC), molecular functions (MF) and biological processes (BP). For CC, the most significantly enriched terms for upregulated genes are *ribosome*, *ATPase complex*, *glial cell projection* and *tertiary granule*, where downregulated terms included *glutamatergic synapse*, *immunological synapse* and *receptor complex* (Fig. 1C). MF terms highlighted *cytokine activity*, *receptor binding* and *protease activity* (Fig. 1D). For BP, the most significantly enriched terms for upregulated genes are *muscle cell proliferation*, *response to activity* and *cytoplasmic translation*. Downregulated terms are *mRNA transcription*, *nuclear transcription* and *nerve development* (Fig. 1E).

A heat map of only the significantly altered genes showed patient-wise elevation of *LRG1*, *CNN2*, *SQSTM1*, *GADD45A*, *FCGR3A*, *CCL3*, *CASP8*, *CTSE*, *TLR2*, *ICAM* and *ITGAM*, alongside consistent reduction of *S100B*, *BCAS1* and *SH2D1A* (Fig. 1F). Over-representation analysis (ORA) was used to identify distinct GO terms, providing further insights into the functional implications of the observed proteomic changes (Fig. 1G). Terms for CC included *oxidoreductase complex*, *presynaptic active zone* and *postsynaptic specialization*. For MF, the top terms are *cytokine activity*, *protein kinase C binding* and *cytokine receptor binding* (Fig. 1H). Terms for BP included T*-cell activation*, *immune response* and *stress response* (Fig. 1I).

### Longitudinal changes of ccfmRNA profile in adult loSMA under risdiplam treatment

**Figure 2:**
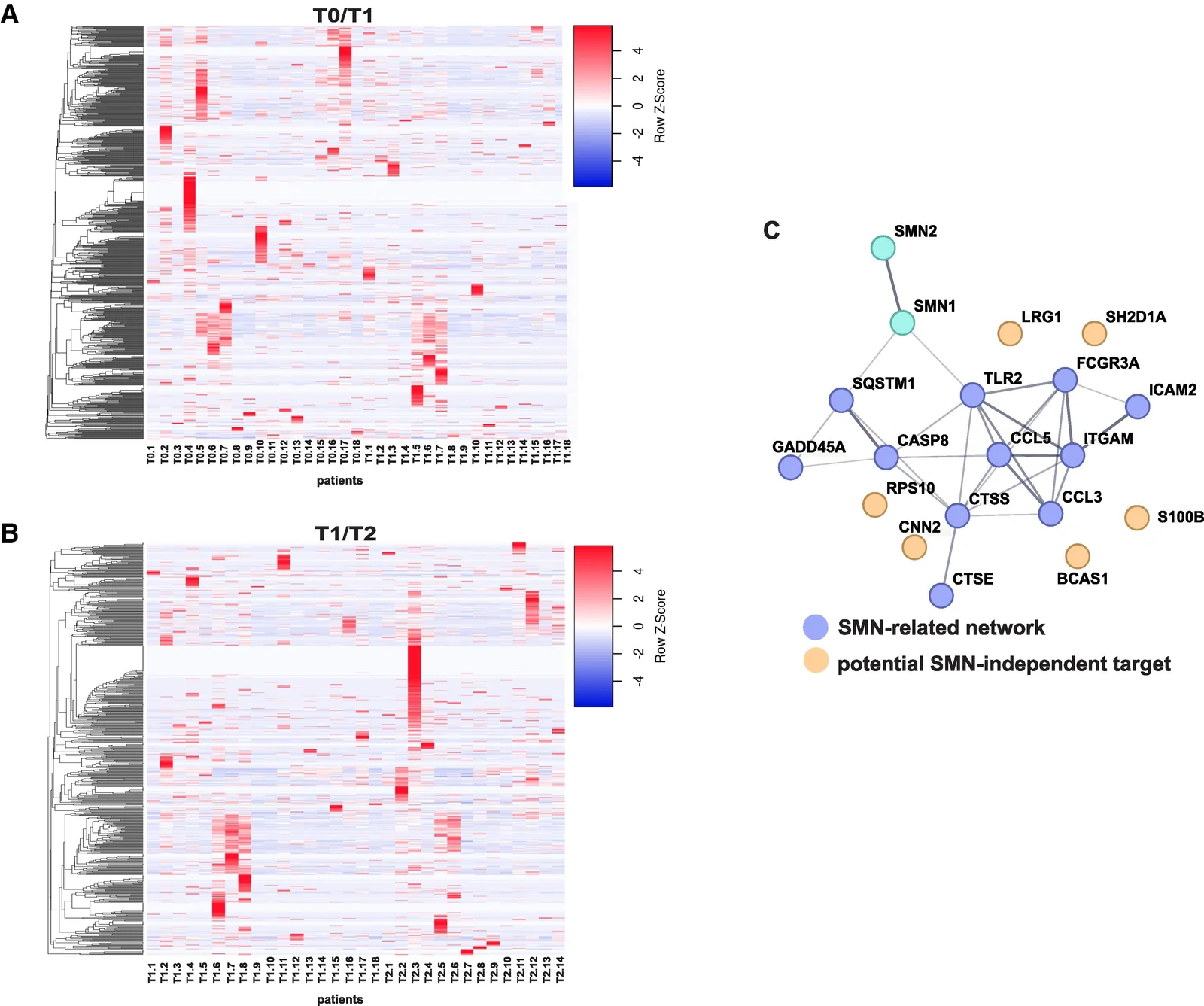
**Changes after long-term risdiplam treatment in loSMA patients.** (**A**) Heat map of all measured transcripts in the serum of T0/T1 patients using directional McQuitty clustering with *Z*-scores plotted for each patient. (**B**) Heat map of all measured transcripts in the serum of T1/T2 patients using directional McQuitty clustering with *Z*-scores plotted for each patient. (**C**) GGI network of upregulated proteins in association with the *SMN1* and *SMN2* genes, showing an SMN-related network and a potential SMN-independent target network. *n* = 17 SMA patients.

Longitudinal analysis of risdiplam-treated adult loSMA patients revealed dynamic transcriptomic changes over time. Hierarchical clustering of serum ccfmRNA profiles showed a substantial molecular shift between baseline (T0) and the first follow-up after treatment initiation (T1) (Fig. 2A), followed by further but less pronounced modulation between T1 and the later follow-up (T2) (Fig. 2B). Both heatmaps showed some patient-wise clustering, indicating that although some patients show similar gene expression profile, there is yet a significant heterogeneity within the patient group.

To explore functional interactions, the differentially expressed genes were mapped onto a gene–gene-interaction (GGI) network, adding *SMN1* and *SMN2* (Fig. 2C). This analysis identified a core SMN-centred cluster comprising *SMN*1 and *SMN2*, connected with modules of immune and stress-related genes (*SQSTM1, TLR2, GADD45A, CASP8, CTSS, CCL5, FCGR3A, intracellular adhesion molecule 2 (ICAM2), ITGAM, CCL3, CTSE, S100B*). Additionally, some genes were not connected to the interaction network (*LRG1*, *SH2D1A*, *RPS10*, *CNN2*, *BCAS1*). The latter group likely represents SMN-independent secondary effectors. The network suggests that the enhanced SMN protein by risdiplam also mitigates downstream molecular dysregulation linked to inflammation and proteostasis.

### Risdiplam reverses elevated immune and proteostasis markers in adult loSMA towards control levels

**Figure 3:**
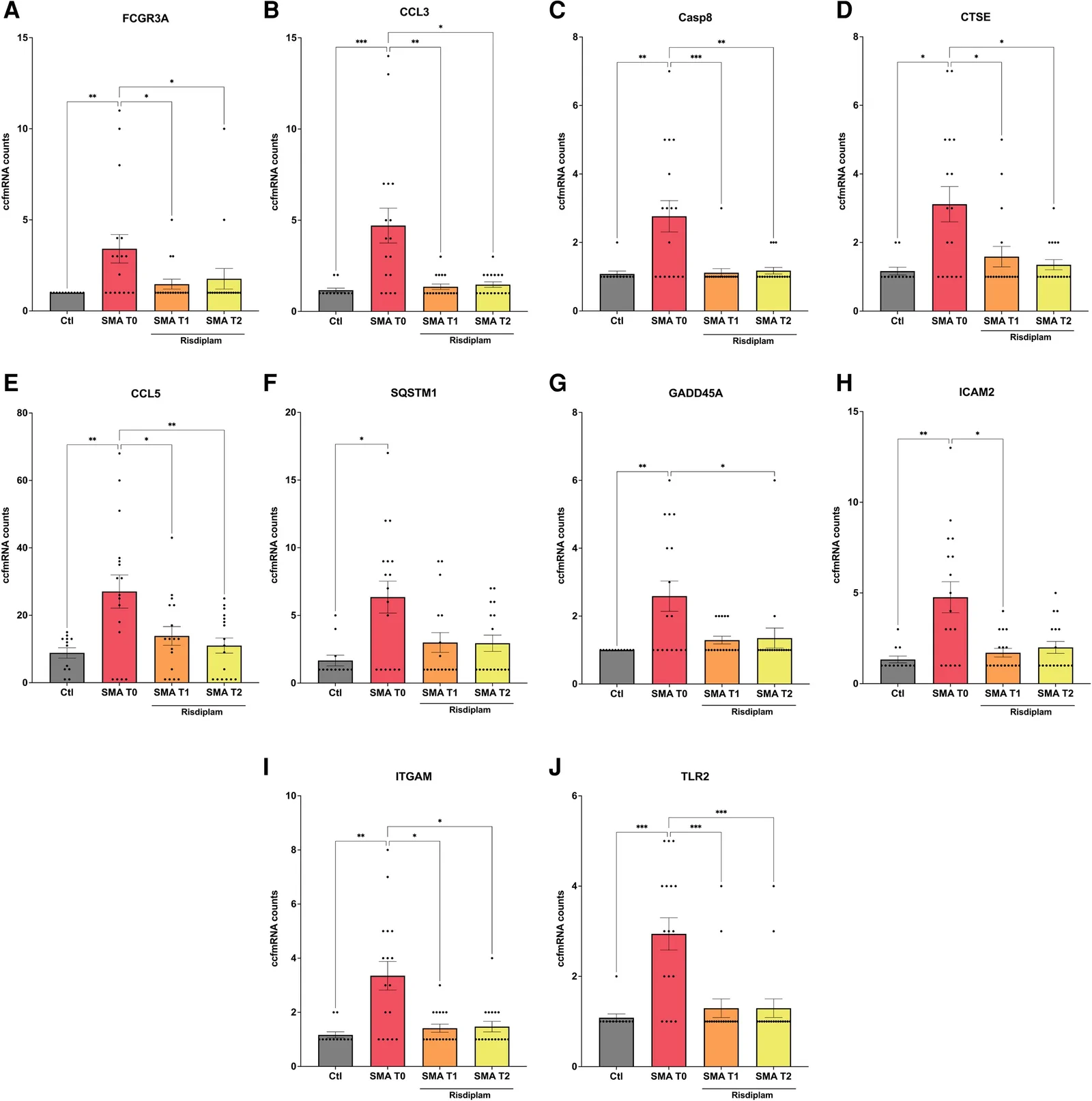
**Risdiplam-altered fcRNA profile in serum of loSMA patients.** (**A**) Adult loSMA patients showed increased *FCGR3A* expression at T0 (Control (Ctl) versus SMA T0, *P* < 0.01), and treatment with risdiplam changed the expression back to baseline (SMA T0 versus SMA T1, *P* < 0.05; SMA T0 versus SMA T2, *P* < 0.05; Kruskal–Wallis). (**B**) Adult loSMA patients showed increased *CCL3* expression at T0 (Ctl versus SMA T0, *P* < 0.001), and treatment with Risdiplam restored expression to baseline (SMA T0 versus SMA T1, *P* < 0.01; SMA T0 versus SMA T2, *P* < 0.05; Kruskal–Wallis). (**C**) In adult loSMA patients increased *CASP8* expression at T0 (Ctl versus SMA T0, *P* < 0.01) was shown, and treatment with risdiplam changed the expression back to baseline (SMA T0 versus SMA T1, *P* < 0.001; SMA T0 versus SMA T2, *P* < 0.01; Kruskal–Wallis). (**D**) In adult loSMA patients, *CTSE* expression was increased at T0 (Ctl versus SMA T0, *P* < 0.05), and treatment with risdiplam restored expression to baseline (SMA T0 versus SMA T1, *P* < 0.05; SMA T0 versus SMA T2, *P* < 0.05; Kruskal–Wallis). (**E**) Adult loSMA patients showed increased *CCL5* expression at T0 (Ctl versus SMA T0, *P* < 0.01), and treatment with risdiplam restored expression to baseline (SMA T0 versus SMA T1, *P* < 0.05; SMA T0 versus SMA T2, *P* < 0.01; ordinary one-way ANOVA). (**F**) Adult loSMA patients showed increased *SQSTM1* expression at T0 (Ctl versus SMA T0, *P* < 0.05; Kruskal–Wallis), and treatment with risdiplam restored expression to baseline. (**G**) Adult loSMA patients showed increased *GADD45A* expression at T0 (Ctl versus SMA T0, *P* < 0.01), and treatment with risdiplam restored expression to baseline (SMA T0 versus SMA T2, *P* < 0.05; Kruskal–Wallis). (**H**) Adult loSMA patients showed increased *ICAM2* expression at T0 (Ctl versus SMA T0, *P* < 0.01), and treatment with risdiplam restored expression to baseline (SMA T0 versus SMA T1, *P* < 0.05). (**I**) Adult loSMA patients showed increased *ITGAM* expression at T0 (Ctl versus SMA T0, *P* < 0.01), and treatment with risdiplam restored expression to baseline (SMA T0 versus SMA T1, *P* < 0.05; SMA T0 versus SMA T2, *P* < 0.05; Kruskal–Wallis). (**J**) Adult loSMA patients showed increased *TLR2* expression at T0 (Ctl versus SMA T0, *P* < 0.001), and treatment with risdiplam restored expression to baseline (SMA T0 versus SMA T1, *P* < 0.001; SMA T0 versus SMA T2, *P* < 0.001; Kruskal–Wallis). *n* = 12 controls, *n* = 17 SMA patients; each datapoint represents an independent measure from a single patient. *P*-value: * < 0.05; ** < 0.01; *** < 0.001.

Targeted quantification of key differentially expressed ccfmRNA transcripts confirmed a consistent reduction of inflammatory and stress-related genes during risdiplam therapy (Fig. 3A–J). At baseline (T0) expression levels of *FCGR3A*, *CCL3*, *CASP8*, *CTSE*, *CCL5*, *SQSTM1*, *GADD45A*, *ICAM2*, *ITGAM* and *TLR2* were significantly elevated (Fig. 3A–J). These genes encode proteins that activate immune and stress-response pathways. Treatment with risdiplam reduced transcript levels to control levels at T1 and T2, indicating a progressive anti-inflammatory effect (Fig. 3A–J). Longitudinal comparisons of the SMA patients using RM design analysis show similar effects (Supplementary Fig. 1A).

### Non-responsive ccfmRNA biomarkers indicate SMN-independent disease features

**Figure 4:**
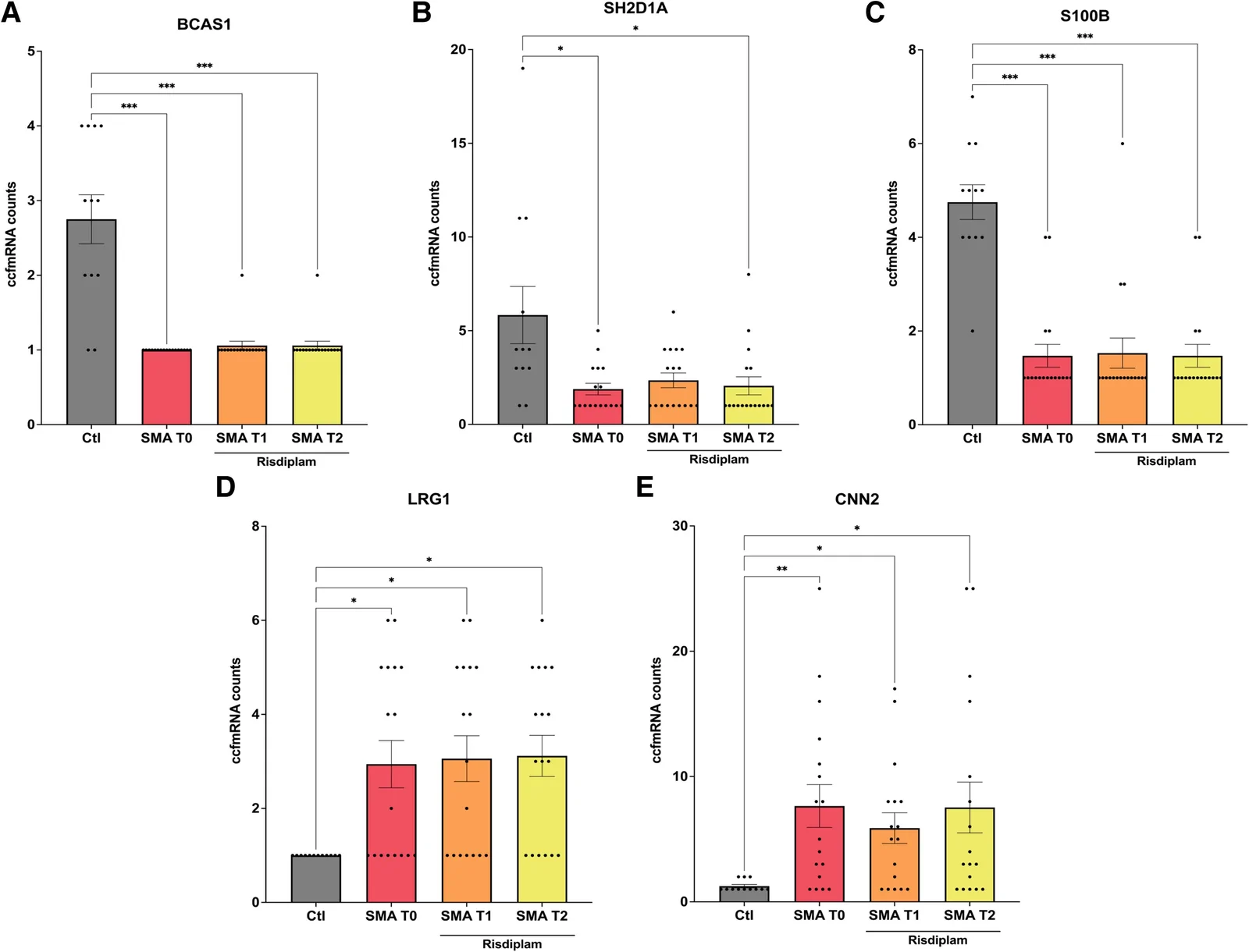
**Risdiplam did not alter the expression pattern of all measured ccfmRNA counts in loSMA patients.** (**A**) Adult loSMA patients showed decreased *BCAS1* expression at T0 (Ctl versus SMA T0, *P* < 0.001), and treatment with risdiplam showed no difference from T0 (Ctl versus SMA T1, *P* < 0.001; Ctl versus SMA T2, *P* < 0.001; Kruskal–Wallis). (**B**) Adult loSMA patients showed decreased *SH2D1A expression* at T0 (Ctl versus SMA T0, *P* < 0.05), and treatment with risdiplam showed no difference from T0 (Ctl versus SMA T2, *P* < 0.05; Kruskal–Wallis). (**C**) Adult loSMA patients showed decreased *S100B* expression at T0 (Ctl versus SMA T0, *P* < 0.001), and treatment with risdiplam showed no difference from T0 (Ctl versus SMA T1, *P* < 0.001; Ctl versus SMA T2, *P* < 0.001; Kruskal–Wallis). (**D**) Adult loSMA patients showed increased *LRG1* expression at T0 (Ctl versus SMA T0, *P* < 0.05), and treatment with risdiplam showed no difference from T0 (Ctl versus SMA T1, *P* < 0.05; Ctl versus SMA T2, *P* < 0.05; Kruskal–Wallis). (**E**) Adult loSMA patients showed increased *CNN2* expression at T0 (Ctl versus SMA T0, *P* < 0.01), and treatment with risdiplam showed no difference from T0 (Ctl versus SMA T1, *P* < 0.05; Ctl versus SMA T2, *P* < 0.05; Kruskal–Wallis). *n* = 12 controls, *n* = 17 SMA patients; each datapoint represents an independent measure from a single patient. *P*-value: * < 0.05; ** < 0.01; *** < 0.001.

Unlike the genes regulated by risdiplam in Fig. 3, some genes were not affected by risdiplam treatment (Fig. 4A–E). Analysis of transcripts revealed reduced expression of *BCAS1*, *S100B* and *SH2D1A* in adult loSMA serum compared to controls. These genes are functionally linked to oligodendrocyte maturation (*BCAS1*), astrocytic calcium signalling (*S100B*) and T-cell/synaptic regulation (*SH2D1A*). In contrast, *LRG1* and *CNN2*, both associated with angiogenesis and cytoskeletal remodelling, were elevated at baseline (Fig. 4A–E).

During risdiplam therapy, transcript levels of *BCAS1, SH2D1A* and *S100B* remained low throughout treatment, while *LRG1* and *CNN2* levels remained elevated (Fig. 4A–E). The differential behaviour of these markers compared with inflammatory genes suggests that risdiplam preferentially normalizes immune activation rather than glial or endothelial signalling.

Longitudinal comparisons of the SMA patients using RM design analysis show similar effects (Supplementary Fig. 1B).

### Phenotype-specific transcriptional signatures distinguish adult loSMA type 2 and loSMA type 3 and reveal common inflammatory modules

**Figure 5:**
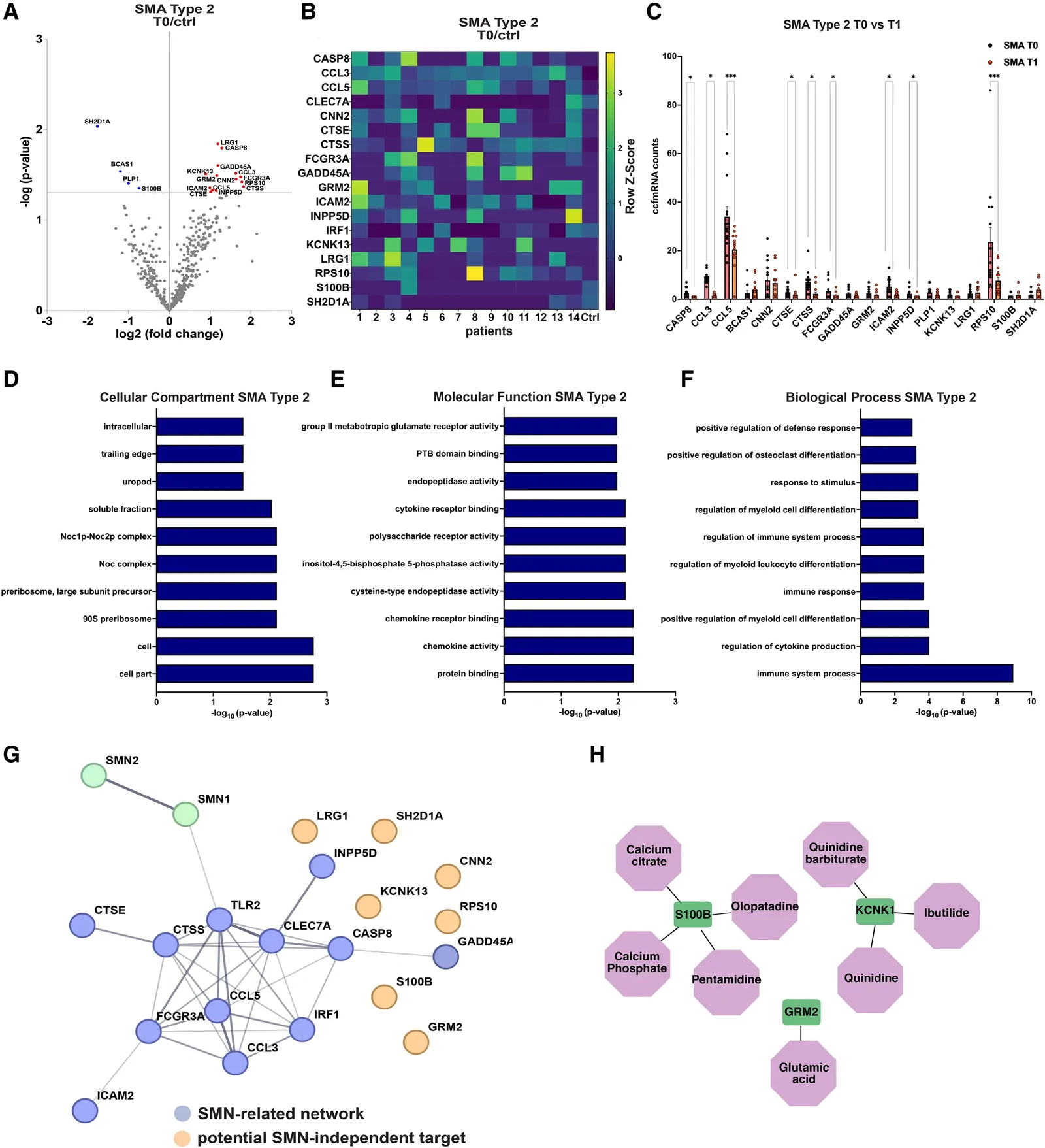
**Changes in ccfmRNA profile under risdiplam treatment in adult loSMA type 2 patients.** (**A**) Volcano plot illustrating the distribution of all transcripts (770). For each gene, the fold change was calculated by dividing the mean transcript abundance in type 2 loSMA patients by the mean transcript abundance in controls. The corresponding *P*-value was calculated from the individual patient values using a two-sided, unpaired Student’s *t*-test. Statistical significance is displayed as −log(*P*) and the horizontal line at −log(*P*) = 1.301 represents the nominal unadjusted significance threshold of *P* = 0.05. Significantly altered proteins (above the horizontal line) are categorized as upregulated or downregulated. (**B**) Heat map of measured transcripts in the serum of adult loSMA type 2 patients. (**C**) mRNA counts of T0 versus T1 of the significantly measured transcripts of adult loSMA type 2 patients (two-way ANOVA). Top ten GO terms of cellular components (**D**), molecular functions (**E**) and biological processes (**F**) identified using ORA. (**G**) String GGI network of significantly regulated transcripts in adult loSMA type 2 in association with the *SMN1* and *SMN2* genes, showing an *SMN*-related network and a potential SMN-independent target network. (**H**) Drug network from the GGI cluster using the NeDREX Cytoscape app. *n* = 12 controls shown as mean, *n* = 14 SMA Type 2 patients; each datapoint represents an independent measure from a single patient. *P*-value: * < 0.05; ** < 0.01; *** < 0.001.

To measure phenotype-specific differences, a heat map and a volcano plot were generated. A volcano plot was constructed to visualize the distribution of all quantified transcripts, using log2 FC plotted against −log10 adjusted *P*-values (|log_2_FC| ≳ 0.5; −log_10_*P* > 1) (Fig. 5A and B).

In adult loSMA type 2, several immune-related genes were markedly upregulated at baseline (*CCL5*, *CCL3*, *CNN2*, *CTSS*, CTSE, *CASP8, ICAM2*, *GADD45A*). Genes related to glial or neuronal support (*S100B*, *BCAS1*, *Proteolipid Protein 1* (*PLP1*)) were comparatively reduced. Risdiplam treatment (T1) normalized most inflammatory transcripts, supporting a robust anti-inflammatory response in this subgroup (Fig. 5C).

GO enrichment for adult loSMA type 2 revealed significant overrepresentation of categories linked to the endoplasmic reticulum, mitochondrial complexes and synaptic compartments, indicating that both stress-response and neuronal-homeostasis pathways are perturbed in this more severe phenotype. Molecular-function analysis emphasized *glutamate receptor activity*, *protein binding* and *PTB-domain binding*. At the same time, biological-process terms included *immune-system process*, *cytokine regulation* and *myelination*, reflecting parallel activation of immune and neuronal programs (Fig. 5D–F).

Additionally, string clustering of significantly regulated transcripts from adult loSMA type 2 patients identified a core SMN-centred cluster comprising *SMN1 and SMN2*, which was connected to modules such as *TLR2*, *CTSS*, *CCL3*, *CASP8*, *CCL5* and others. Additionally, some genes were not connected to the interaction network, including *LRG1*, *SH2D1A*, *RPS10*, *CNN2*, *Potassium 2 Pore Domain Channel Subfamily K Member 13* (*KCNK13*)*, Glutamate Metabotropic Receptor 2* (*GRM2*) and *S100B*. The latter group likely represents *SMN*-independent secondary effectors (Fig. 5G).

To identify potential drug targets, the significantly altered genes were used in an *in silico*-based target identification approach employing the network medicine platform NeDREX.^22^

Network-based drug identification analysis identified multiple approved therapeutic compounds targeting these proteins, suggesting their potential for pharmacological intervention. For instance, pentamidine or olopatadine was identified as a drug target for *S100B* (Fig. 5H).

**Figure 6:**
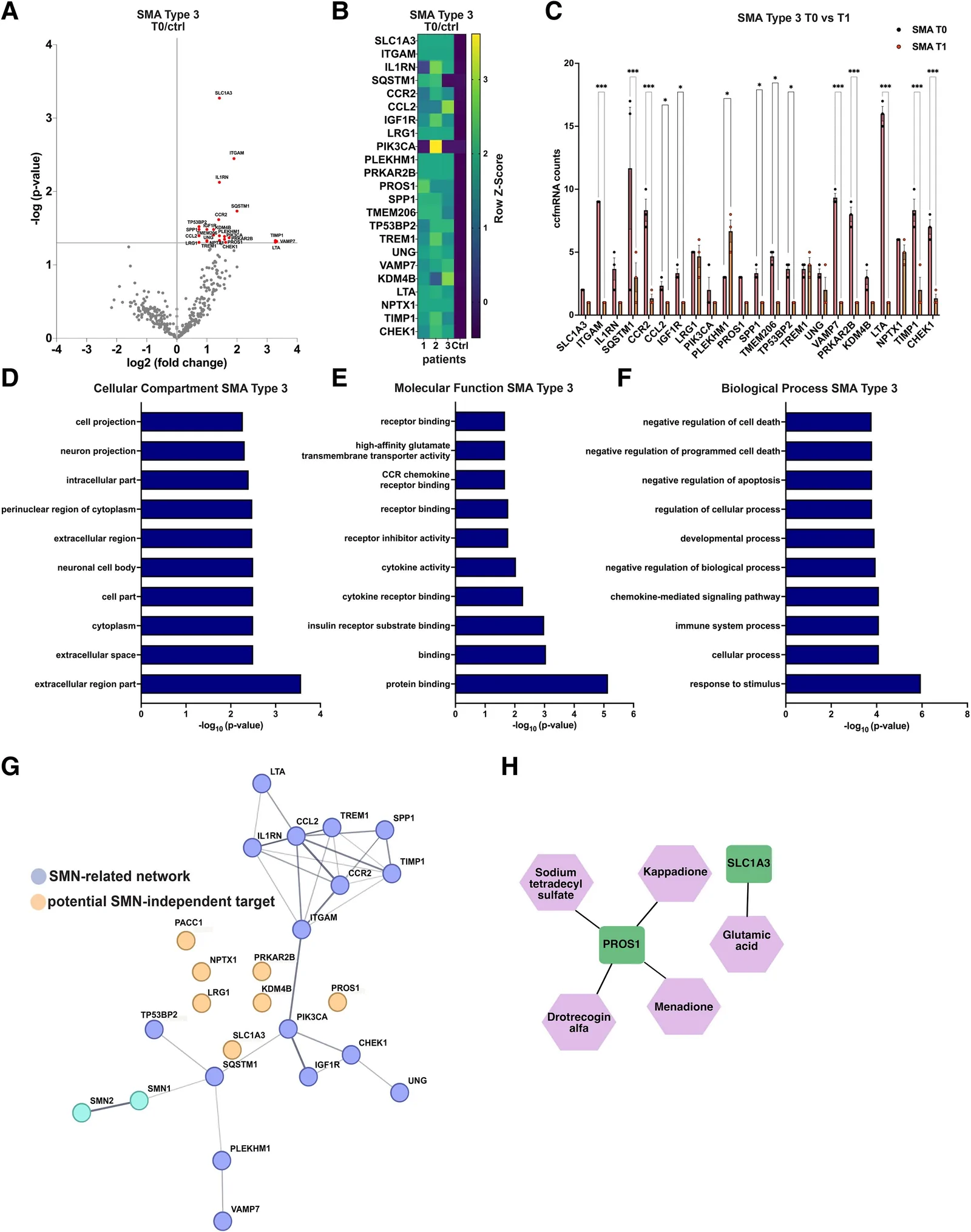
**Changes in ccfmRNA profile under risdiplam treatment in loSMA type 3 patients.** (**A**) Volcano plot illustrating the distribution of all transcripts (770). For each gene, the fold change was calculated by dividing the mean transcript abundance in type 3 loSMA patients by the mean transcript abundance in controls. The corresponding *P*-value was calculated from the individual patient values using a two-sided, unpaired Student’s *t*-test. Statistical significance is displayed as −log(*P*) and the horizontal line at −log(*P*) = 1.301 represents the nominal unadjusted significance threshold of *P* = 0.05. Significantly altered proteins (above the horizontal line) are categorized as upregulated or downregulated. (**B**) Heat map of measured transcripts in the serum of adult loSMA type 2 patients. Z-scores are plotted for each patient. (**C**) ccfmRNA counts of T0 versus T1 of the significantly measured transcripts of adult loSMA type 3 patients (two-way ANOVA). Top ten GO terms of cellular components (**D**), molecular functions (**E**) and biological processes (**F**) identified using ORA. (**G**) String GGI network of significantly regulated transcripts in adult loSMA type 2 in association with the *SMN1* and *SMN2* genes, showing an SMN-related network and a potential *SMN*-independent target network. (**H**) Drug network from the GGI cluster using the NeDREX Cytoscape app. *n* = 12 controls shown as mean, *n* = 3 SMA Type 3 patients; each datapoint represents an independent measure from a single patient. *P*-value: * < 0.05; ** < 0.01; *** < 0.001.

In adult loSMA type 3, baseline alterations were milder but followed a similar pattern. Risdiplam treatment again reduced *ITGAM*, *SQSTM1*, *C-C Motif Chemokine Receptor 2* (*CCR2*), *CCL2*, *Vesicle Associated Membrane Protein 7* (*VAMP7*), *Secreted Phosphoprotein 1* (*SPP1*), *Protein Kinase CAMP-Dependent Type II Regulatory Subunit Beta* (*PRKAR2B)*, *Lymphotoxin Alpha* (*LTA)*, *TIMP Metallopedidase Inhibitor 1* (*TIMP1*) and *Checkpoint Kinase 1* (*CHEK1*) expression (Fig. 6A–C). Differentially expressed genes included *Solute Carrier Family 1 Member 3* (*SLC1A3*), *ITGAM*, *Interleukin 1 Receptor Antagonist* (*IL1RN*), *SQSTM1* and *CCL2*. GO terms showed dominant annotation terms in *extracellular region*, *cytokine activity* and *immune response*, suggesting persistent but attenuated inflammatory engagement (Fig. 6D–F).

Additionally, string clustering of significantly regulated transcripts of adult loSMA type 3 patients identified a core SMN-centred cluster comprising SMN*1*, *SMN2*, connected with modules such as *SQSTM1*, *Insulin-like Growth Factor 1 Receptor* (*IGF1R*), *ITGAM*, *CCR2*, *SPP1*, *CCL2* and others. Additionally, some genes were not connected to the interaction network including *Protein S* (*PROS1*), *LRG1*, *Proton Activated Chloride Channel 1* (*PACC1*), *SLC1A3*, *Lysine Demethylase 4B* (*KDM4B*) and *PRKAR2B*. The latter group likely represents *SMN*-independent secondary effectors (Fig. 6G).

To identify potential drug targets, the significantly altered genes were used in an *in silico*-based target identification approach employing the network medicine platform NeDREX.^22^

Network-based drug identification analysis identified multiple approved therapeutic compounds targeting these proteins, suggesting their potential for pharmacological intervention. For instance, menadione or kappadione was identified as a drug target for PROS1 (Fig. 6H).

### Exploratory comparison of ccfmRNA profiles between non-sitter and sitter adult loSMA patients

Due to the ongoing redefinition and refinement of SMA phenotype classification, we additionally stratified patients into non-sitters and sitters, rather than using the conventional SMA type 2 and type 3 categories. This classification did not reveal any additional differences. Overall, non-sitters showed a profile comparable to that of individuals classified as SMA type 2, whereas sitters displayed a pattern similar to that observed in SMA type 3 patients (Supplementary Figs 2 and 3).

However, direct comparison of non-sitters versus sitters at both T0 and T1 revealed pronounced differences. To complement the subtype-based analyses, we performed an exploratory comparison of serum ccfmRNA profiles according to functional status, stratifying adult loSMA patients into non-sitters and sitters at baseline (T0). Volcano plot analysis revealed a distinct transcriptional pattern (Supplementary Fig. 4A). Among the transcripts contributing to this pattern were *Leukocyte Immunoglobulin Like Receptor B4* (*LILRB4), MutS Homologue 2* (*MSH2), Sialic Acid Binding Ig Like Lectin 1* (*SIGLEC1*)*, Replication Protein A1* (*RPA1*)*, Interferon Regulatory Factor 1* (*IRF1*)*, Myocyte Enhancer Factor 2C* (*MEF2C*)*, C-Type Lectin Domain Containing 7a* (*CLEC7A*)*, ST8 Alpha-N-Acetyl-Neuraminide Alpha-2,8-Sialyltransferase 6* (*ST8SIA6*)*, APC Regulator Of Wnt Signalling Pathway* (*APC*)*, CD69, Aldehyde Dehydrogenase 1 Family Member L1* (*ALDH1L1*)*, Transmembrane Proteine 204* (*TMEM204), Transglutaminase 1* (*TGM1*)*, Calsyntenin 1* (*CLSTN1*)*, PLP1, Solute Carrier Family 17 Member 6* (*SLC17A6*)*, SWI/SNF Related BAF Chromatin Remodelling Complex Subunit D1* (*SMARCD1*)*, Autophagy Related 5* (*ATG5*)*, Presenilin 2* (*PSEN2*)*, Cyclin 1* (*CCNI*)*, T-Box Brain Transcription Factor 1* (*TBR1*)*, Baculoviral IAP Repeat Containing 5* (*BIRC5*)*, Non-SMC Condensin I Complex Subunit H (NCAPH*)*, CD3 Epsilon Subunit Of T-Cell Receptor Complex* (*CD3E*) and *Trafficking From ER To Golgi Regulator*, which were all downregulated in non-sitters compared to sitters. Heat map visualization confirmed that these transcripts were generally more abundant in sitters than in non-sitters, although inter-individual heterogeneity remained evident within both groups (Supplementary Fig. 4B). Over representation analysis of the transcripts differing between non-sitters and sitters identified enriched GO categories related to chromosomal and microtubule-associated cellular compartments, kinase- and receptor-related molecular functions and biological processes linked to lymphocyte activation, leukocyte activation, hematopoiesis and immune-system development (Supplementary Fig. 4C–E). A similar exploratory comparison was performed after treatment initiation at T1. The volcano plot again showed a predominance of transcripts with lower abundance in non-sitters relative to sitters (Supplementary Fig. 4F). Among the transcripts contributing to this pattern were *CLSTN1, Paired Immunoglobin Like Type 2 Receptor Alpha* (*PILRA*)*, Poly(ADP-Ribose) Polymerase 2* (*PARP2*)*, Mannosidase Alpha Class 2B Member 1* (*MAN2B1*)*, Histone Deacetylase 2* (*HDAC2*)*, Activating Transcription Factor 3* (*ATF3), Charged Multivesicular Body Protein 1A* (*CHMP1A*)*, Cathepsin D* (*CTSD*)*, PLP1, CD68, SET Domain Bifurcated Histone Lysine Methyltransferase 1* (*SETDB1*)*, BCL2 Like 11* (*BCL2L11), Cardiotrophin Like Cytokine Factor 1* (*CLCF1), Caspase 2* (*CASP2*)*, CD19, P21 (RAC1) Activated Kinase 1* (*PAK1*)*, HUS1 Checkpoint Clamp Component* (*HUS1*)*, BCL2 Antagonist/Killer 1* (*BAK1), Gap Junction Protein Beta 1* (*GJB1*)*, Phospholipase C Gamma 2* (*PLCG2*)*, MYC Target 1* (*MYCT1), SEL1L Adaptor Subunit Of SYVN1 Ubiquitin Ligase* (*SEL1L*)*, CD163, SET Domain Containing 1B, Histone Lysine Methytransferase* (*SETD1B*) and *Fatty Acid Binding Protein 5* (*FABP5*). The heat map at T1 again demonstrated a group-wise clustering tendency, with sitters retaining comparatively higher expression of many differentially abundant transcripts than non-sitters (Supplementary Fig. 4G). ORA at T1 yielded a broadly similar functional profile, with enrichment of cellular compartment terms linked to chromosomal and microtubule-associated structures (Supplementary Fig. 4H), molecular functions related to binding and receptor activity (Supplementary Fig. 4I) and biological processes centred on immune cell activation, lymphocyte differentiation, haematopoiesis and negative regulation of apoptosis (Supplementary Fig. 4J). Taken together, these exploratory analyses suggest that functional status in adult loSMA is associated with distinct serum ccfmRNA patterns at both baseline and follow-up, with non-sitters showing an overall relative reduction in several immune-, glial- and signalling-associated transcripts compared with sitters. These findings are consistent with functional-status-associated differences in immune-related and cellular organization pathways.

### Transcriptional adaptation in nusinersen-to-risdiplam switcher patients reveals overlapping and distinct regulatory pathways

**Figure 7:**
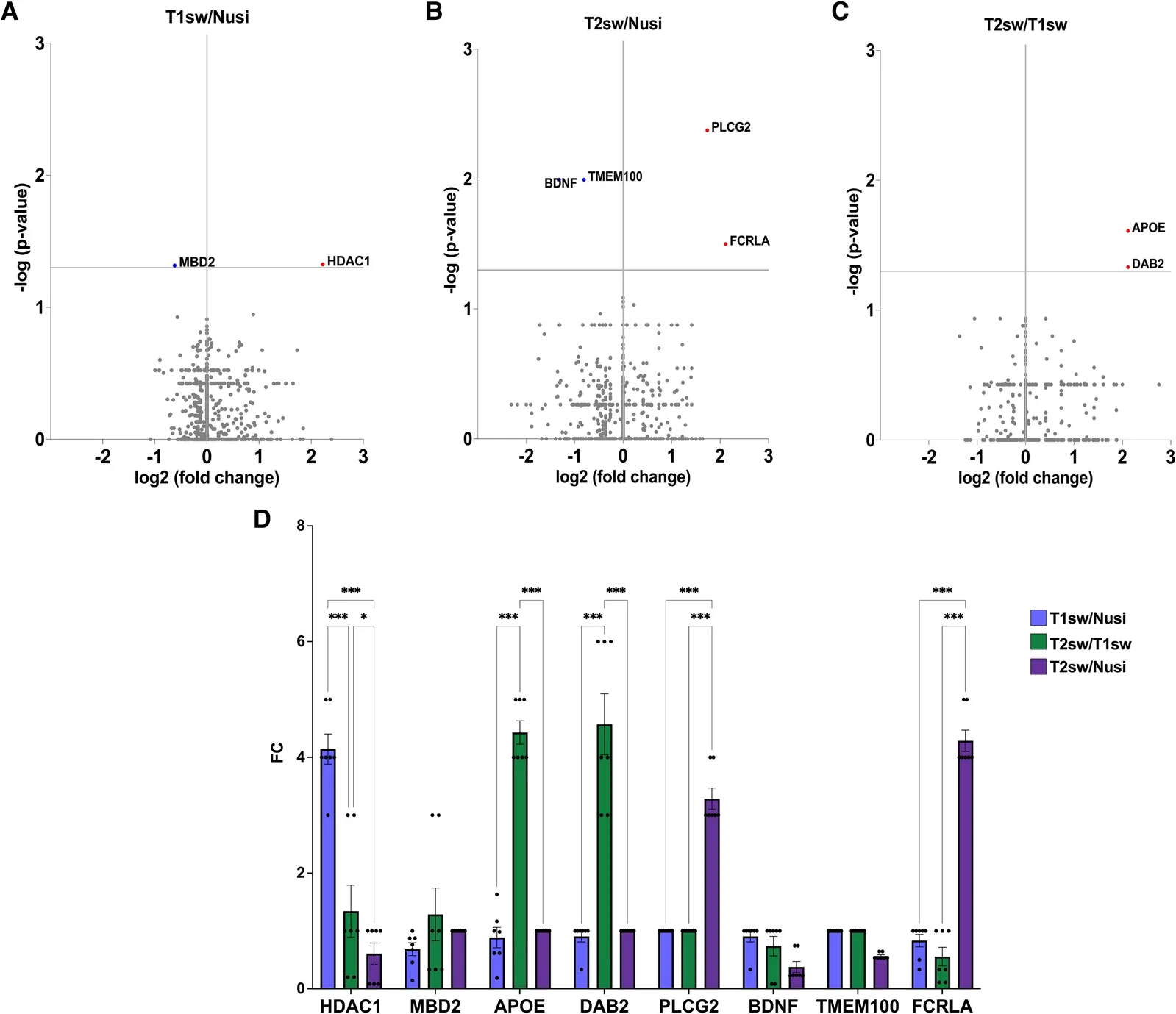
**Changes in ccfmRNA profile in adult loSMA patients after switching from nusinersen to risdiplam.** (**A–C)** Volcano plot illustrating the distribution of all transcripts (770). For each gene, the fold change was calculated by dividing the mean transcript abundance in loSMA patients after switching by the mean transcript abundance in controls. The corresponding *P*-value was calculated from the individual patient values using a two-sided, unpaired Student’s *t*-test. Statistical significance is displayed as −log(*P*) and the horizontal line at −log(*P*) = 1.301 represents the nominal unadjusted significance threshold of *P* = 0.05. Significantly altered proteins (above the horizontal line) are categorized as upregulated (red) or downregulated (blue), showing changed genes: *MBD2, HDAC1, APOE, DAB2, BDNF, TMEM100, PLCG2, FCRLA*. (**D**) Graph showing the expressed transcript for each condition. *HDAC1* is reduced comparing T1sw/Nusi with T2sw/T1sw (*P* < 0.001) and T1sw/Nusi and T2sw/Nusi (*P* < 0.001). *APOE* is increased comparing T1sw/Nusi with T2sw/T1sw (*P* < 0.001) and decreased between T2sw/T1sw and T2sw/Nusi (*P* < 0.001). *PLCG2* is increased comparing T1sw/Nusi with T2sw/Nusi (*P* < 0.001) and T2sw/T1sw and T2sw/Nusi (*P* < 0.001). *FCRLA* is increased when comparing T1sw/Nusi with T2sw/Nusi (*P* < 0.001) and T2sw/T1sw and T2sw/Nusi (*P* < 0.001) (two-way ANOVA, Tukey’s multiple comparison test). *n* = 7 SMA switchers (sw/Nusi); each datapoint represents an independent measure from a single patient. *P*-value: * < 0.05; ** < 0.01; *** < 0.001.

Volcano plots comparing transcriptional changes across time points in adult switcher (sw) patients (those who transitioned from nusinersen to risdiplam) showed distinct modulation patterns between the stages (Fig. 7A–D). A volcano plot was constructed to visualize the distribution of all quantified proteins, using log2 FC plotted against −log10 adjusted *P*-values (|log_2_FC| ≳ 0.5; −log_10_*P* > 1) (Fig. 7A–C).

Between nusinersen and early risdiplam treatment (T1 sw/Nusi), a small subset of transcripts changed significantly, most notably *HDAC1* and *Methyl-CpG Binding Domain Protein 2* (*MBD2*) (Fig. 7A and D). These genes are associated with phagocytic signalling and chromatin remodelling, respectively, suggesting that switching therapy may influence immune-metabolic homeostasis.

At later time points (T2 sw versus Nusi and T2 sw versus T1 sw; Fig. 7B–D), additional genes became regulated, including *Apolipoprotein E (APOE)*, *DAB Adapter Protein 2 (DAB2)*, *PLCG2*, *Brain-Derived Neurotrophic Factor* (*BDNF*), *Transmembrane Protein 100* (*TMEM100*) and *Fc Receptor Like A* (*FCRLA)*, which are central to lipid metabolism, vesicular trafficking and B-cell receptor signalling.

Quantitative comparison of normalized counts across all switcher conditions confirmed that several transcripts were significantly increased following the switch from nusinersen to risdiplam (Fig. 7D). Notably, *BDNF* and *TMEM100* upregulation imply a compensatory activation of neurotrophic and vascular-stabilizing mechanisms, potentially reflecting systemic readouts of neuronal plasticity and improved cellular communication.

The observed gene clusters collectively point towards therapy-specific transcriptional trajectories: risdiplam induces gradual immune normalization, while nusinersen evokes stronger activation of neuronal plasticity and lipid-associated repair pathways.

## Discussion

In this exploratory study, we analysed ccfmRNA in serum samples from adult loSMA type 2 and type 3 patients and healthy controls, including individuals treated with risdiplam and six patients who had switched from nusinersen to risdiplam.

Our work builds on a previous ccfmRNA serum analysis in loSMA type 2 and 3 patients treated with nusinersen using the NanoString neuropathology panel.^19^ That study identified four genes, *Adhesion Molecule With Ig Like Domain 1* (*AMIGO1)*, *Carbonic Anhydrase 2* (*CA2*), *CCL5* and *TLR2*, with altered transcript counts in adult loSMA versus healthy controls, showing phenotype- and response-specific patterns. Notably, these changes persisted after 6 months of nusinersen, despite robust SMN enhancement.^19^ The study thus suggested that these transcripts reflect SMN-independent pathways and may serve as adjunctive therapeutic targets rather than direct pharmacodynamic markers of nusinersen.

In the exploratory study presented here, serum samples from adult loSMA patients (baseline levels, T0) showed elevated expression of innate immune mediators, chemokines and stress-response genes, including *CCL3*, *CCL5*, *TLR2*, *CASP8*, *SQSTM1* and *GADD45A*, alongside downregulation of glial and synaptic support transcripts, such as *S100B*, *BCAS1* and *SH2D1A*. This pattern could support the hypothesis that that loSMA in adults is accompanied by chronic peripheral immune activation and cellular stress beyond motor neurons, involving glial and vascular compartments, as well as muscle and other organs.^23-31^ This inflammatory transcript pattern could also be consistent with NF-κB-associated signalling, which has recently been implicated in immune dysregulation and post-treatment systemic pathology in SMA,^32^ however, NF-κB pathway activity was not directly assessed in the present study. Circulating cell-free mRNAs are interesting in this context because they are highly stable, generally enriched in the nervous system, and if originating from the nervous system, then from genes involved in synaptic function, neuronal differentiation and stress responses.^15,17,18,33^

Risdiplam treatment resulted in marked reductions of diverse inflammatory and stress-related transcripts, bringing several of them back toward control levels. Genes such as *CCL3*, *CCL5*, *TLR2*, *CASP8*, *SQSTM1* and *GADD45A* showed a clear trend towards normalization, suggesting that *SMN2* splicing modulation could possibly dampen systemic immune activation and cellular stress in adult loSMA. These findings complement preclinical data demonstrating that SMN upregulation improves cellular homeostasis and mitochondrial function and align with transcriptomic changes reported during nusinersen therapy.^19,34^ However, not all ccfmRNAs followed the same direction of change under risdiplam. A subset of transcripts remained persistently dysregulated even after prolonged treatment, including *BCAS1*, *S100B* and *SH2D1A,*^35,36^ as well as *LRG1* and *CNN2,* which are linked to angiogenesis and cytoskeletal remodelling.^37^ These genes can be associated with oligodendrocyte maturation,^38,39^ astrocytic signalling,^40-42^ immune regulation and vascular remodelling^43,44^ and their persistent dysregulation in turn could suggest the presence of SMN-independent or only partially SMN-sensitive pathways that are not fully rescued by risdiplam. Such residual signatures could possibly contribute to the persistent weakness and fatigability often reported by treated adults and highlight the importance of further research in a larger cohort as well as of adjunct therapeutic strategies targeting inflammation, glial dysfunction, metabolism or vascular pathology in adult loSMA patients who initiate treatment later in the disease course.^45-49^

A central aspect of our exploratory study is the comparison between adult loSMA type 2 and type 3. Clinically, these subtypes differ in age at onset, motor milestones and degree of irreversible motor neuron loss.^50-52^ In general terms, adult loSMA type 2 shows more pronounced changes, in line with its clinically more severe phenotype, whereas loSMA type 3 patients display a qualitatively similar but attenuated dysregulation, however the type 3 group is small and needs further investigation with a more balanced sample size in the future. For example, adult loSMA type 2 patients showed a stronger inflammatory and stress-associated signature, indicated by higher expression of *CCL2*, *CXCL10*, *IL1B* and *HSPA1A*, while loSMA type 3 patients exhibited a milder but overlapping pattern. This is similar to the findings, in which *CA2* was particularly associated with loSMA type 3, and *CCL5* and *AMIGO1* changes were more prominent in adult loSMA type 2 and in non-responders.^19^ The current data suggest that adult loSMA type 2 and type 3 may share some overlapping but also different transcriptional features with potentially larger effect sizes in the more clinically severe subgroup. Confirmations of such differences will require substantially larger cohorts. An exploratory stratification by functional status showed that non-sitters and sitters also differed in their serum ccfmRNA profiles, with enrichment of immune-, apoptosis- and cytoskeleton-associated pathways at both T0 and T1. However, direct comparisons of sitter T0 versus controls and non-sitter T0 versus controls did not reveal substantially broader differences than the type 2 versus type 3 analyses, which likely reflects the considerable overlap between clinical subtype and functional status in our cohort, where most type 2 patients were non-sitters, as well as the overall limited subgroup size. Nevertheless, these differences should be considered when searching for biomarker fingerprints or designing adjunctive therapies.

One major question in the current SMA care is how to identify patients who will benefit most from a given disease-modifying therapy and, conversely, who will remain non-responders despite SMN restoration. In a recently published study, we found that transcripts such as *CA2*, *CCL5*, *AMIGO1* and *TLR2* differed between responders and non-responders, even though they did not correlate directly with motor scores and did not change under nusinersen.^19^ This pattern was interpreted as a ‘background signature’ that might mark patients with a more unfavourable biology, for example, more pronounced inflammatory activation or impaired plasticity, who respond less well to SMN-targeted therapy. Our ccfmRNA profile data extend this concept by showing that a similar situation may occur at the levels of circular and linear transcripts, and with a different SMN-enhancing drug. Although our adult loSMA cohort was not powered to define a robust ‘responder ccfmRNA panel’, the fact that certain transcripts remain differentially expressed under risdiplam, whereas others are closer to control levels, suggests that baseline or early-treatment ccfmRNA profiles might help to identify patients with more SMN-independent pathology who are at risk of limited functional benefit. Future studies combining ccfmRNA measurements with established markers such as SMN protein, neurofilaments and quantitative motor scores will be needed to test this hypothesis systematically.^32^

Patients who transitioned from nusinersen to risdiplam represented a particularly informative subgroup. Clinically, switching from nusinersen to risdiplam is performed for reasons of side effects such as regular lumbar puncture, perceived suboptimal response, patient’s convenience or clinical decision. In our dataset, the nusinersen-to-risdiplam switcher displayed distinct transcriptional dynamics compared with treatment-naïve risdiplam patients. Early after switching, we observed changes in epigenetic regulators such as *HDAC1*^53,54^ and *MBD2*,^55^ followed by later modulation of genes involved in lipid handling (e.g. *APOE* & *DAB2*), neuronal plasticity (e.g. *BDNF*)^56^ and vascular stabilization (e.g. *TMEM100*). The observed temporal trajectory suggests that switching between SMN-targeted therapies may induce a molecular re-equilibration phase, plausibly reflecting mechanistic and biodistribution differences between intrathecal and oral delivery. Although these observations are based on a small subset of seven individuals and must be interpreted with great caution, they support the emerging view that the sequencing and combination of SMN-enhancing therapies may be biologically meaningful and that molecular monitoring could help to optimize clinical decision-making.

Overall, our findings support serum ccfmRNA profiling as a promising ‘liquid biopsy’ approach in loSMA. The clear discrimination between loSMA and control samples, longitudinal normalization of many signals under treatment, subtype-specific signatures and therapy-switching dynamics indicate that ccfmRNA can capture disease biology and therapeutic response. Moreover, the presence of persistent non-normalized transcripts suggests that ccfmRNA readouts may help generate hypotheses about residual biology not fully normalized by SMN-enhancing therapy. The main value of this approach at present is therefore exploratory. These data complement prior reports on ccfmRNAs transcripts in nusinersen-treated patients^19^ and align with the broader biomarker literature, which emphasizes the need for multimodal approaches in SMA.^34,57,58^ With respect to the serum biomarker candidates identified, it should be noted that circulating biomarker levels may be influenced by confounding factors such as age, renal function, body mass index and physical activity. Adjustment for these potential confounders was not performed in the present study but will be essential in future validation studies using larger patient cohorts.

## Conclusion

We show that adult loSMA types 2 and 3 are associated with distinct serum ccfmRNA signatures, that risdiplam treatment is accompanied by partial but not complete normalization of these signatures, and that some transcripts remain persistently dysregulated, reminiscent of the SMN-independent ccfmRNA changes previously described under nusinersen.^19,34^ These findings strengthen the concept that part of the systemic disease burden in adult loSMA is not directly corrected by SMN restoration and that ccfmRNAs can help to identify such residual pathophysiological modules. In the long term, integrating ccfmRNA profiling into multimodal biomarker strategies may support more precise prediction of therapy response, inform decisions about switching or combining SMN-enhancing drugs, and help prioritize adjunctive treatments. However, the present results are preliminary and require validation in larger prospective cohorts before conclusions regarding the subtype classification, treatment switching, response prediction, or clinical decision-making can be drawn.

## Supplementary Material

fcag352_Supplementary_Data

## Data Availability

Data that support the findings of this study are available upon reasonable request from the corresponding author.
