# Supplementary material for: Serum circulating cell-free messenger RNA profile response to risdiplam treatment in adult patients with late-onset spinal muscular atrophy
Source: Brain Commun. 2026 Sep 25;8(5):fcag352. doi: 10.1093/braincomms/fcag352 (PMC13613217; doi:10.1093/braincomms/fcag352)
Supplement: Supplementary file 1 [file fcag352_supplementary_data.zip › Supplementary_materials.pdf]

Original Article

Serum Circulating Cell-free Messenger RNA Profile Response to Risdiplam-Treatment in Adult Patients with Late-Onset Spinal Muscular Atrophy

Short title: Late-onset SMA cf-mRNA and Risdiplam

Markus Leo[1, *], Linda-Isabell Schmitt[1], Kai Christine Liebig[1], Stefanie Hezel[1], Svenja Neuhoff[1], Andreas Roos[2,3,4], Tobias Ruck[2,3], Christoph Kleinschnitz[1], Tim Hagenacker[1]

[1]Department of Neurology, Center for Translational Neuro- and Behavioural Sciences (C-TNBS), University Medicine Essen, 45147 Essen, Germany

[2]Ruhr University Bochum, BG University Hospital Bergmannsheil, Department of Neurology, Bochum, Germany

[3]BG University Hospital Bergmannsheil, Heimer Institute for Muscle Research, Bochum, Germany

[4]Department of Neuropediatrics, Centre for Neuromuscular Disorders in Children, University Children's Hospital Essen, University of Duisburg-Essen, 45147 Essen, Germany.

*Corresponding author: markus.leo@uk-essen.de

Keywords: circulating mRNA, cfRNA, biomarker, neuromuscular disorders, liquid biopsy

Longitudinal alterations of circulating cell-free messenger RNA (ccfmRNA) profiles in serum of late-onset spinal muscular atrophy (loSMA) patients during risdiplam treatment

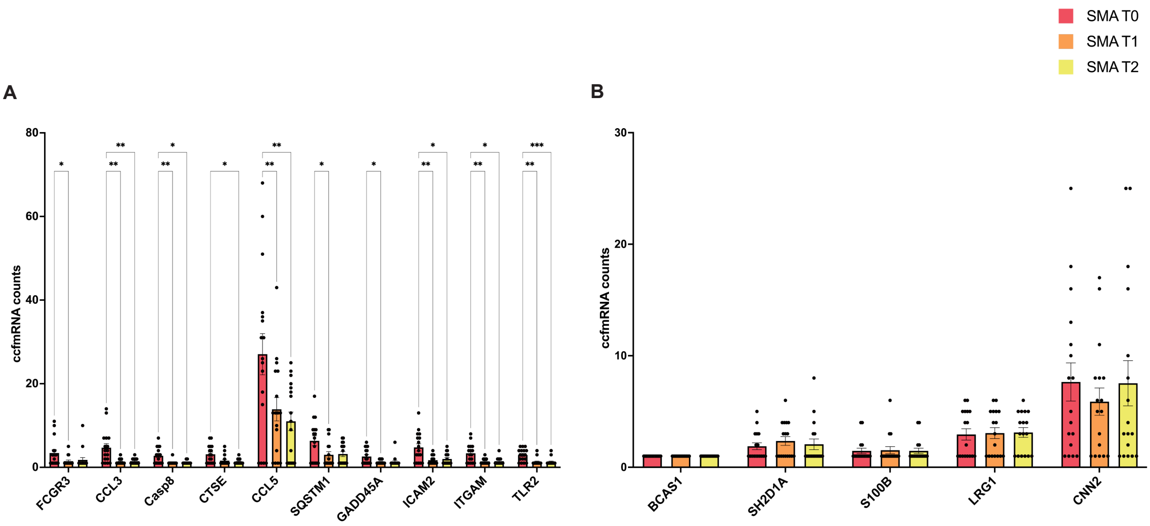

Supplementary Figure 1 Longitudinal alterations of circulating cell-free messenger RNA (ccfmRNA) profiles in serum of late-onset spinal muscular atrophy (loSMA) patients during risdiplam treatment. A Adult loSMA patients showed reduced expression of *Fc Gamma Receptor III A* (*FCGR3A)* risdiplam treatment (SMA T0 vs SMA T1, p<0.05). Adult loSMA patients showed reduced *C-C Motif Chemokine Ligand III* (*CCL3)* expression after treatment with risdiplam (SMA T0 vs SMA T1, p<0.01; SMA T0 vs SMA T2 p<0.01). Adult loSMA patients showed increased *Caspase 8* (*Casp8)* expression at T0, and treatment with risdiplam reduced the expression back to baseline (SMA T0 vs SMA T1, p<0.01; SMA T0 vs SMA T2, p<0.05). In adult loSMA patients, *Cathepsin E* (*CTSE*) expression was increased at T0 and treatment with risdiplam decreased the expression (SMA T0 vs SMA T1, p<0.05). Adult loSMA patients showed increased *C-C Motif Chemokine Ligand 5* (*CCL5)* expression at T0, and treatment with risdiplam showed a reduced *CCL5* expression (SMA T0 vs SMA T1, p<0.01; SMA T0 vs SMA T2, p<0.01). Adult loSMA patients showed increased *Sequestosome 1* (*SQSTM1)* expression at T0, and treatment with risdiplam reduced the expression (SMA T0 vs SMA T1, p<0.05). Adult loSMA patients showed increased *Growth Arrest and DNA Damage Inducible Alpha* (*GADD45A)* expression at T0, and treatment with risdiplam reduced the expression (SMA T0 vs SMA T1, p<0.05). Adult loSMA patients showed increased *Intracellular Adhesion Molecule 2* (*ICAM2)* expression, and treatment with risdiplam reduced the expression (SMA T0 vs SMA T1, p<0.01; SMA T0 vs SMA T2, p<0.05). Adult loSMA patients showed increased *Integrin Subunit Alpha M* (*ITGAM*) expression at T0, and treatment with risdiplam reduced the expression (SMA T0 vs SMA T1, p<0.01; SMA T0 vs SMA T2, p<0.05). Adult loSMA patients showed increased *Toll Like Receptor 2* (*TLR2*) expression at T0 and treatment with risdiplam changed the expression (SMA T0 vs SMA T1, p<0.01; SMA T0 vs SMA T2, p<0.001) (Two-way ANOVA Tukey's multiple comparison test; Geisser-Greenhouse's epilepson). **B** Adult loSMA patients no change in the expression

of *Brain Enriched Myelin Associated Protein 1 (BCAS1)*, *SH2 Domain Containing 1A (SH2D1A)*, *Calcium Binding Protein A1 S100 (S100B)*, *Leucine Rich Alpha-2-Glycoprotein 1 (LRG1)* and *Calponin 2 (CNN2)* after treatment with risdiplam (SMA T0 vs SMA T1, p>0.05; SMA T0 vs SMA T2, p>0.05) (Two-way ANOVA Tukey's multiple comparison test; Geisser-Greenhouse's epilepson). n=17 SMA patients. Each datapoint represents an independent measure from a single patient. P-value: *<0.05; **<0.01; ***<0.001.

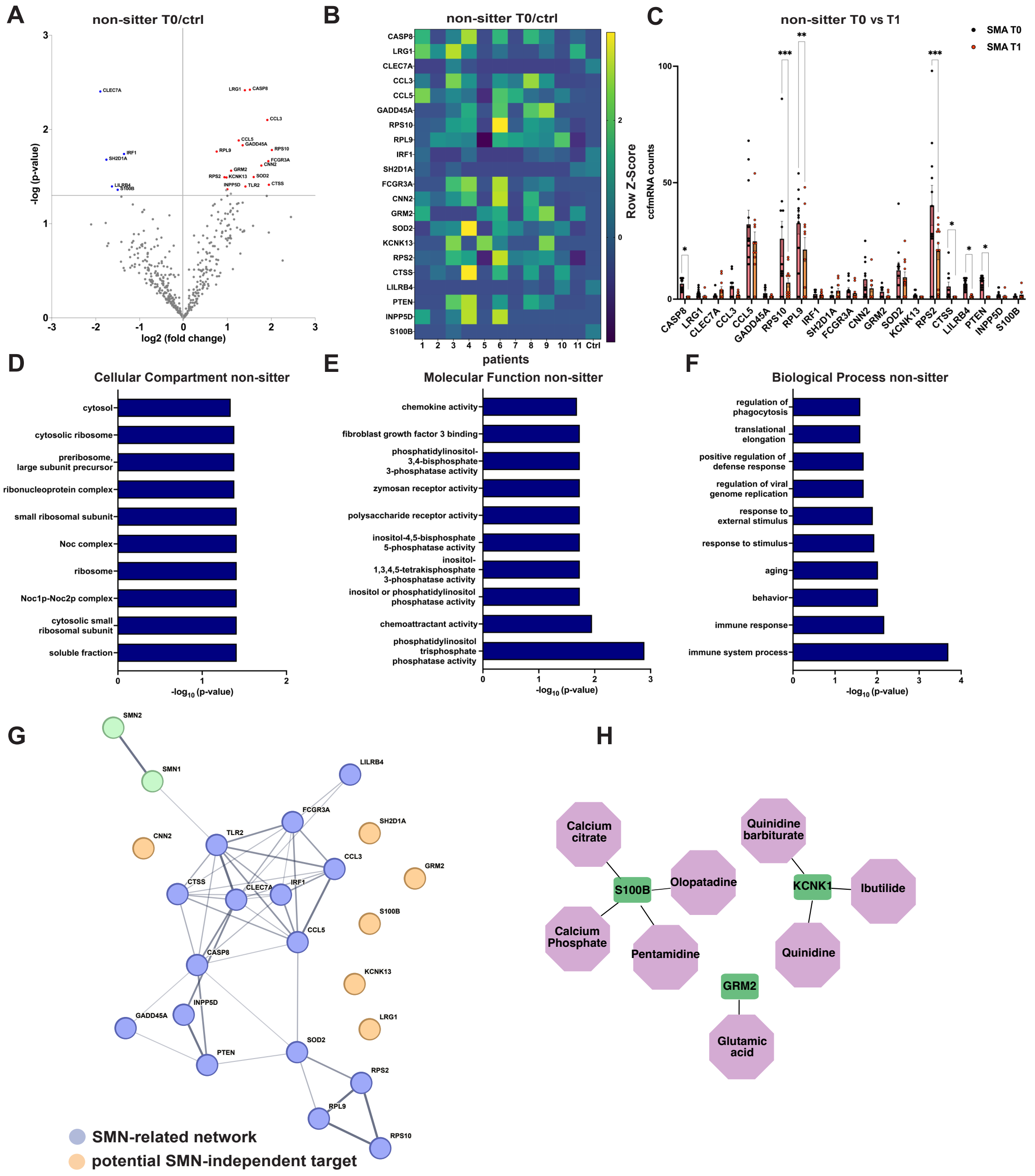

Supplementary Figure 2 Changes of circulating cell-free messenger RNA (ccfmRNA) profile under risdiplam treatment in adult late-onset spinal muscular atrophy (loSMA) non-sitter patients. A Volcano plot illustrating the distribution of all transcripts (770). For each gene, the fold change was calculated by dividing the mean transcript abundance in untreated non-sitter loSMA patients by the mean transcript abundance in controls. Corresponding p-value was calculated from the individual patients values using a two-sided, unpaired Student's t-test. Statistical

significance is displayed as -log(p) and the horizontal line at -log(p) = 1.301 represents the nominal unadjusted significance threshold of p = 0.05. Significantly altered proteins (above the horizontal line) are categorized as upregulated (red) or downregulated (blue). **B** Heat map of measured transcripts of the serum of adult loSMA type 2 patients. **C** mRNA counts of T0 vs T1 of the significantly measured transcripts of adult loSMA type 2 patients (Two-way ANOVA Tukey's multiple comparison test). **D-F.** Top ten Gene Ontology (GO) terms of Cellular Components (G), Molecular Functions (H), and Biological Processes (I) identified using ORA. **G** String Gene-Gene-Interaction (GGI) network of significantly regulated transcripts in adult loSMA type 2 in association with the *survival of motoneuron* (*SMN) 1* and *SMN2* genes, showing an *SMN*-related network and a potential *SMN*-independent target network. **H** Drug network from the GGI cluster using them NeDREX Cytoscape app. n=12 controls shown as mean, n=11 SMA Type 2 patients. Each datapoint represents an independent measure from a single patient. P-value: *<0.05; **<0.01; ***<0.001.

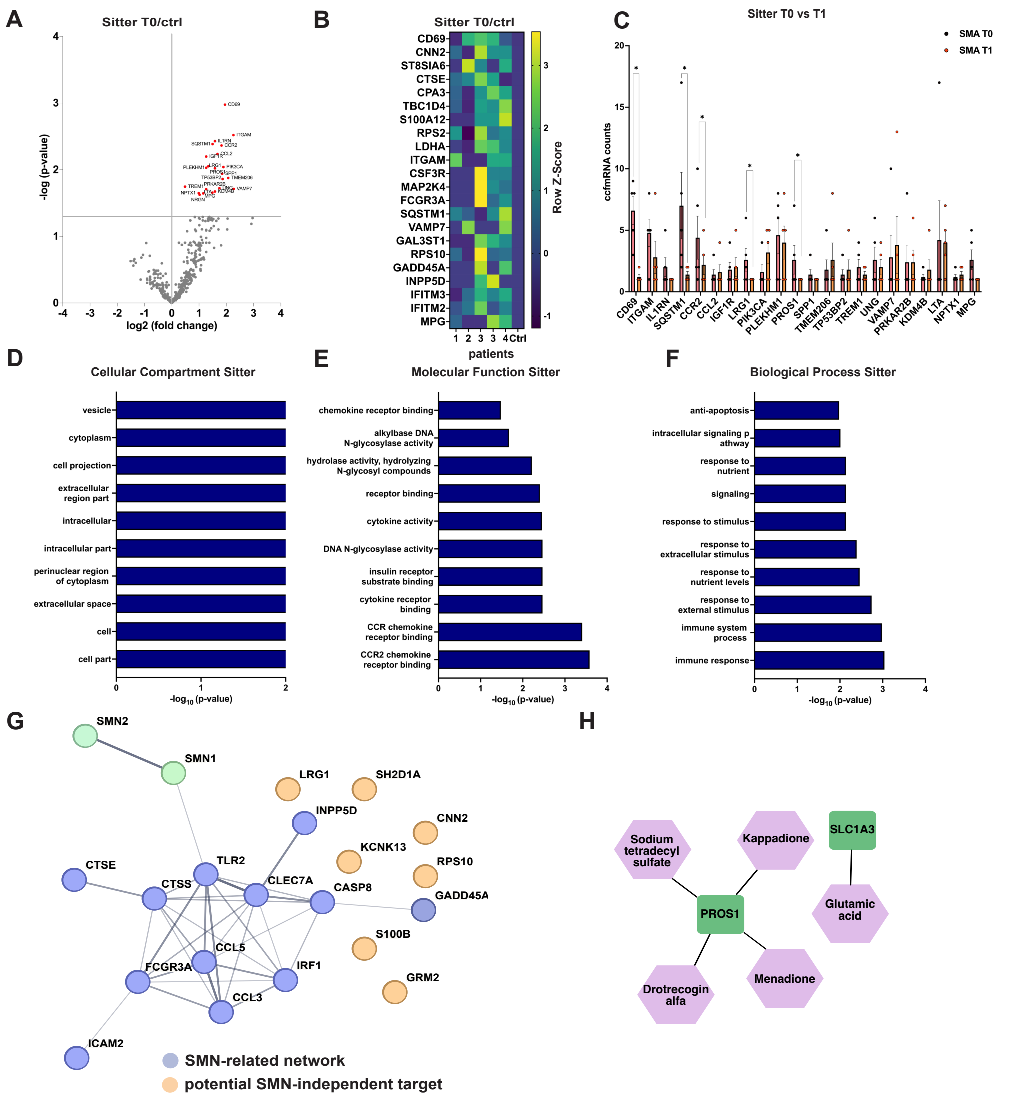

Supplementary Figure 3 Changes of circulating cell-free messenger RNA (ccfmRNA) profile under risdiplam treatment in late-onset spinal muscular atrophy (loSMA) sitter patients. A Volcano plot illustrating the

distribution of all transcripts (770). For each gene, the fold change was calculated by dividing the mean transcript abundance in untreated sitter loSMA patients by the mean transcript abundance in controls. Corresponding p-value was calculated from the individual patients values using a two-sided, unpaired Student's t-test. Statistical significance is displayed as -log(p) and the horizontal line at -log(p) = 1.301 represents the nominal unadjusted significance threshold of p = 0.05. Significantly altered proteins (above the horizontal line) are categorized as upregulated (red) or downregulated (blue). **B** Heat map of measured transcripts of the serum of adut loSMA type 2 patients. Z-scores are plotted for each patient. **C** CcfmRNA counts of T0 vs T1 of the significantly measured transcripts of adult loSMA type 3 patients (Two-way ANOVA Tukey's multiple comparison test). **D-F.** Top ten Gene Ontology (GO) terms of Cellular Components (G), Molecular Functions (H), and Biological Processes (I) identified using Over Representation Analysis (ORA). **G** String Gene-Gene-Interaction (GGI) network of significantly regulated transcripts in adult loSMA type 2 in association with the *survival of motoneuron* (*SMN) 1* and *SMN2* genes, showing an *SMN*-related network and a potential *SMN*-independent target network. **H** Drug network from the GGI cluster using the NeDREX Cytoscape app. n=12 controls shown as mean, n=5 SMA Type 3 patients. Each datapoint represents an independent measure from a single patient. P-value: *<0.05; **<0.01; ***<0.001.

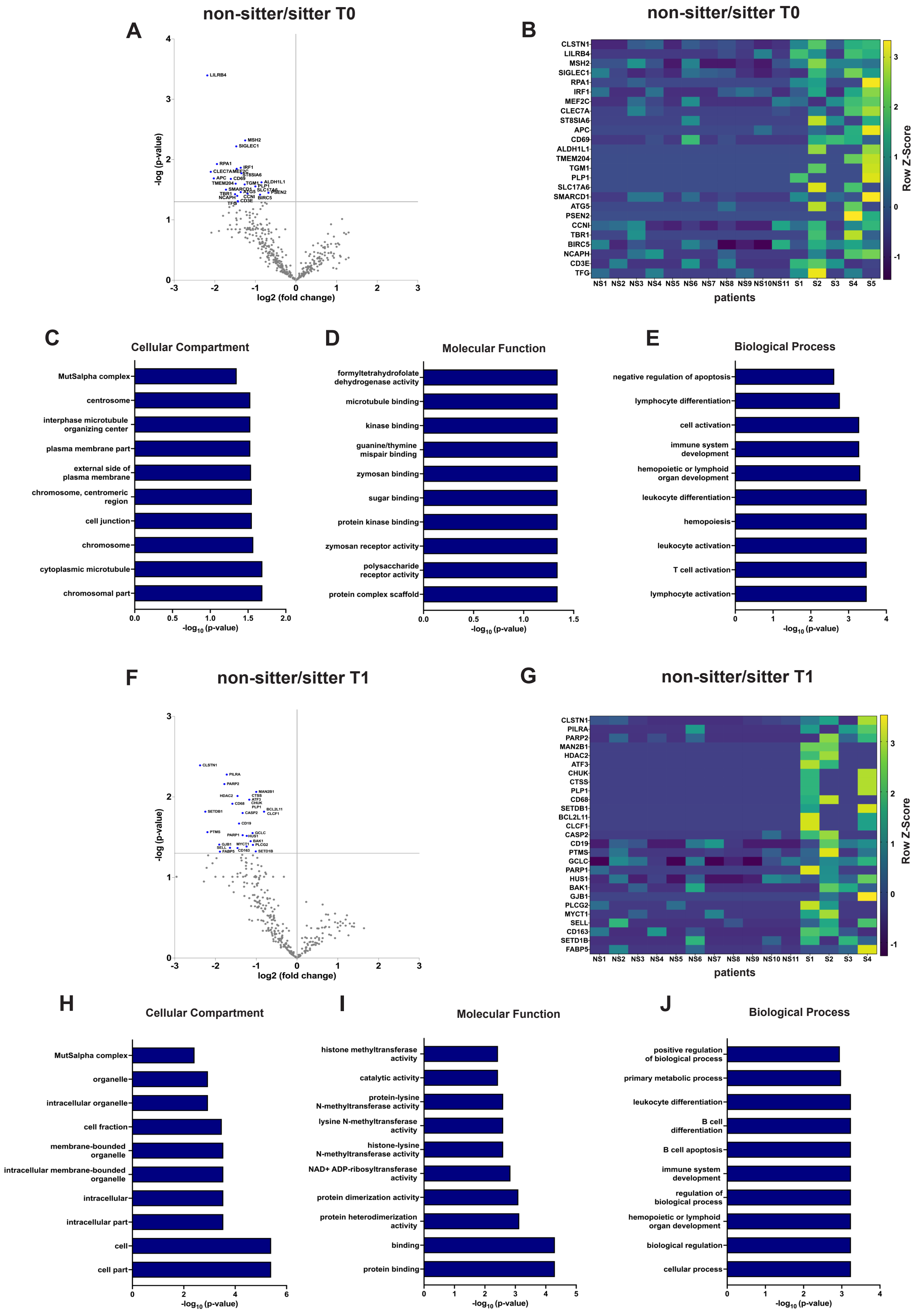

Supplementary Figure 4 Comparison of serum circulating cell-free messenger RNA (ccfmRNA) profiles between non-sitter and sitter adult late-onset spinal muscular atrophy (loSMA) patients. A Volcano plot

showing transcripts (770) differentially abundant in non-sitter versus sitter adult loSMA patients at baseline (T0;). For each gene, the fold change was calculated by dividing the mean transcript abundance in non-sitter loSMA patients by the mean transcript abundance sitter loSMA patients. Corresponding p-value was calculated from the individual patients values using a two-sided, unpaired Student's t-test. Statistical significance is displayed as -log(p) and the horizontal line at -log(p) = 1.301 represents the nominal unadjusted significance threshold of p = 0.05. Log2 fold change is plotted against –log10(p-value). Negative fold change indicates lower transcript abundance in non-sitters/sitters, whereas positive fold change indicates higher abundance in non-sitters. **B** Heat map of significantly altered transcripts in non-sitter and sitter adult loSMA patients at baseline (T0). Z-scores are shown for each individual patient. **C–E** Top ten enriched Gene Ontology (GO) terms identified by Over Representation Analysis (ORA) for the significantly altered transcripts in the comparison of non-sitters/sitters at T0, shown for cellular component (E), molecular function (F), and biological process (G). **F** Volcano plot showing transcripts (770) differentially abundant in non-sitter versus sitter adult loSMA patients at baseline (T1). For each gene, the fold change was calculated by dividing the mean transcript abundance in non-sitter T1 loSMA patients by the mean transcript abundance sitter T1 loSMA patients. Corresponding p-value was calculated from the individual patients values using a two-sided, unpaired Student's t-test. Statistical significance is displayed as -log(p) and the horizontal line at -log(p) = 1.301 represents the nominal unadjusted significance threshold of p = 0.05. Log2 fold change is plotted against –log10(p-value). Negative fold change indicates lower transcript abundance in non-sitters/sitters, whereas positive fold change indicates higher abundance in non-sitters. G Heat map of significantly altered transcripts in non-sitter and sitter adult loSMA patients at baseline (T1). Z-scores are shown for each individual patient. **H–J** Top ten enriched Gene Ontology (GO) terms identified by Over Representation Analysis (ORA) for the significantly altered transcripts in the comparison of non-sitters/sitters at T1, shown for cellular component (E), molecular function (F), and biological process (G). n = 11 non-sitters and n = 5 sitters at T0; longitudinal comparisons were performed in n = 11 non-sitters and n = 5 sitters at T1. *p < 0.05, **p < 0.01, ***p < 0.001.
